# Validation of a Questionnaire to Analyze Teacher Training in Inclusive Education in the Area of Physical Education: The CEFI-R Questionnaire

**DOI:** 10.3390/ijerph20032306

**Published:** 2023-01-28

**Authors:** Jorge Rojo-Ramos, María Mendoza-Muñoz, Santiago Gómez-Paniagua, Miguel Ángel García-Gordillo, Ángel Denche-Zamorano, Jorge Pérez-Gómez

**Affiliations:** 1 Physical Activity for Education, Performance and Health (PAEPH) Research Group, Faculty of Sports Sciences, University of Extremadura, 10003 Cáceres, Spain; 2 Research Group on Physical and Health Literacy and Health-Related Quality of Life (PHYQOL), Faculty of Sport Sciences, University of Extremadura, 10003 Caceres, Spain; 3 Departamento de Desporto e Saúde, Escola de Saúde e Desenvolvimento Humano, Universidade de Évora, 7004-516 Évora, Portugal; 4 BioẼrgon Research Group, University of Extremadura, 10003 Cáceres, Spain; 5 Universidad Autónoma de Chile, Talca 3467987, Chile; 6 Social Impact and Innovation in Health (InHEALTH) Research Group, Faculty of Sport Sciences, University of Extremadura, 10003 Cáceres, Spain; 7 Health, Economy, Motricity and Education (HEME) Research Group, Faculty of Sport Sciences, University of Extremadura, 10003 Cáceres, Spain

**Keywords:** inclusive education, diversity, perceptions, physical education, CEFI-R

## Abstract

Inclusive education is a right that has captured the attention of public institutions, researchers, and teaching professionals around the world. The beliefs and perceptions of teachers are a fundamental axis in knowing the state of these professionals regarding this ethical principle, allowing them to develop and implement different strategies. This study aims to explore the reliability and factor structure of the Evaluation of Teachers’ Preparation for Inclusion (CEFI-R) questionnaire among physical education teachers. The sample consisted of 789 Spanish in-service teachers who completed the questionnaire to assess their readiness for inclusion in this subject. Exploratory and confirmatory factor analyses as well as reliability testing were carried out. A factor structure with four dimensions (conception of diversity, methodology, support, and community participation) was obtained. These dimensions consisted of 17 items with good and excellent goodness-of-fit values. In addition, a high reliability was obtained (Cronbach’s Alpha = 0.71−0.93). Therefore, the CEFI-R could be considered a valid and reliable tool to analyze physical education teachers’ perceptions of their preparedness for inclusive education.

## 1. Introduction

Since the 1990s, inclusive education has become one of the major topics of discussion in the field of education [1], generating a large amount of scientific literature in order to characterize the current state in which different nations are in order to develop strategies for its implementation and improvement [2]. This idea has been theorized and investigated in a variety of fields, including psychology, pedagogy, and education, and it has been in line with initiatives to respect diversity in today’s schools [3]. Inclusive education, by definition, ensures that students have access to all the support and services they need to participate fully in general education activities and curriculum [4]. An inclusive school is therefore an environment in which all children should learn together, wherever possible, regardless of any difficulties or differences they may have [5]. In this sense, adopting a socio-ecological perspective on the interplay between students’ capacities and environmental needs is necessary for inclusive education, which places emphasis on the need for educational systems to adapt to and reach all students [6].

As a result, significant adjustments to training and values have been made to better prepare teachers for inclusive and diverse classrooms [7]. The relationship between teachers’ perceptions and attitudes toward inclusion and successful inclusive practices has been supported by prior research, suggesting that teachers’ beliefs about the nature of disability and their ability have an impact on how they perceive their roles and responsibilities with these students, as well as how they shape their practice [8,9]. Additionally, it was discovered that all of the children in the classroom benefited from the inclusive practices used by teachers who adhered to inclusive ideologies and epistemologies [10]. Therefore, pre-service training and professional development programs now include more knowledge on inclusive education in an effort to spread positive perceptions that will lead to effective inclusive educational practices [11]. However, despite this current trend of improving training in inclusion, research has produced a consensus on the general perception that teachers perceive themselves to be poorly trained to develop their professional activity in an inclusive classroom [12].

In addition, students with disabilities have limited access to extracurricular activities [13], and they are six times less likely to report high levels of self-efficacy in physical education (PE) than peers who receive high marks in school-based PE [14]. In this regard, PE is an essential context for personal development due to that childhood is the time when lifestyle patterns are formed, and actions taken throughout adolescence may have an impact on future habits of physical activity, health, and subjective well-being [15]. Pupils from various functional backgrounds are included in PE classes, which increases the number of students in the class while simultaneously improving physical function and developing and reinforcing motor skills [16]. Studies in the field of PE have found that the presence of students with disabilities has no negative influence on the performance of their peers [17], but that sharing PE sessions with a student with a disability creates positive attitudes towards the inclusion of students with special needs [18]. Examples of an inclusive teaching approach with potential participation benefits include adapting traditional sports and offering optional activities at suitable skill levels [19]. Nevertheless, the literature shows that few studies report participatory benefits of inclusive PE-oriented interventions; however, individually tailored programs for students with disabilities have been developed [20].

Similarly, there are several barriers that affect the perceptions of PE teachers regarding inclusive education. The most common research that focuses on the study of PE teachers’ gender, generally presents different results, with most of them showing non-significant differences [21,22]. In addition, class size, time restraints, curriculum requirements, a lack of training and professional development, concerns for students’ safety, potential harm to peers, perceptions of the type and severity of impairments, and student behavior were all listed as barriers [23,24,25]. By contrast, peer approval [26], teachers’ external or internal motivation for inclusion [27], teaching social skills [28], working with support teachers [29], parents [30], and other teachers [31] are some of the facilitators that have a positive impact on PE teachers’ perceptions of educational inclusion. It has also been observed that teachers’ practical experiences in inclusive classrooms during their initial training have a positive impact on their attitudes and perceptions of what it is like to be a teacher in an inclusive classroom [32], in a way that reduces their anxiety about including pupils with special needs in sessions and increases their confidence in these contexts. However, the age of the teacher does not seem to be a clear predictor of the teacher’s perceptions of inclusion [33], despite some research indicating that younger and less experienced teachers have more favorable attitudes [21,34] either through greater exposure to inclusive policies or greater readiness for inclusion.

Consequently, developing tools to assess teachers’ perceptions of their readiness for inclusion in the area of PE is therefore an urgent necessity [35], as the future physical activity and health levels of students with disabilities may be negatively influenced by the results of these practices [36]. Current tools and scales that assess inclusion in the area of PE do not focus specifically on teachers’ perceptions of their training for inclusion, but rather assess overall needs for inclusion [37], the promotion of this right in the subject [38], their attitudes towards the inclusion of students with special education needs [39], or teacher self-efficacy [40]. In this line, the Evaluation of Teachers’ Preparation for Inclusion (CEFI-R) [41,42] could be considered as a free, easy, and quick to administer instrument with which to assess teachers’ perceptions of their training for inclusion. However, it has never been validated in the context of PE. Therefore, the purpose of this research is to present the validity, reliability, and structure of the CEFI-R in PE teachers from different schools in the Community of Extremadura, Spain. In this way, contextualizing teacher training needs will enable institutions to develop inclusive teaching programs applicable to both future and in-service teachers.

## 2. Materials and Methods

### 2.1. Participants

Seven hundred eighty-nine teachers from centers throughout the Autonomous Community of Extremadura’s primary and secondary schools made up the sample (Spain). Table 1 displays their characteristics, with a mean experience of 15.33 years (Sd = 10.01). A non-probability convenience sampling technique was used to select the participants [43].

It should be noted that according to the latest data provided by the National Institute of Statistics (INE), women represent more than 50% in all educational areas except physical education, where they represent only 17.1%. It should also be noted that the province of Badajoz has 437 primary and secondary schools (66.6%), while Cáceres has 291 (33.4%). All this explains the large differences in the variables gender and province of the center in Table 1.

### 2.2. Instruments

Gender, center province, University of Extremadura studies, age, and years of experience were the five sociodemographic questions that were created to describe the sample.

The CEFI-R, or Evaluation of Teachers’ Preparation for Inclusion [41,42], is made up of 19 items in total that are divided into four dimensions (Appendix A). The dimensions that conform the questionnaire were extracted from two reference publications, the Profile of Inclusive Teachers [44], published by European Agency for Development in Special Needs Education, and the Index for Inclusion [45], written by Booth and Ainscow. After the initial validation analysis [41], 4 of the 5 dimensions originally proposed were confirmed: (1) conception of diversity; (2) methodology; (3) support; and (4) community participation. The first dimension is composed of 5 items which examine teachers’ perceptions about diversity, the setting and type of their education, as well as towards diversity education policy. The second factor is linked to the creation and implementation of an inclusive curriculum and consists of 5 items. In addition, the third dimension shows 4 items about the teacher’s conception of the role of the support teacher. Finally, the last factor includes 5 categories that evaluate the cooperation of all educational actors. Each item of the CEFI-R is composed of a 4-point Likert-type scale, due to that it allows the elimination of neutral responses by characterizing them positively or negatively, ranging from 1, corresponding to “Strongly disagree”, to 4, corresponding to “Strongly agree”. For the analysis, it was necessary to transpose the indirect items to match each of the dimensions. In the initial investigation, the results yielded a consistence value of 0.79, which was >0.70 for each of the four factors [46].

### 2.3. Procedure

Teachers at the public primary and secondary schools in Extremadura were sent an email to obtain the sample. The Ministry of Education and Employment of the Regional Government of Extremadura (Spain) was utilized to gain access to the schools’ email accounts. An informed consent form, information about the study’s objectives, and a link to the questionnaire for the teachers who wanted to participate in the study were all included in that email.

Thus, the Google Forms tool was used for participants to fill in both the sociodemographic data and the CEFI-R, thus trying to reduce material costs, obtaining a higher return and delivery rate [47]. After data recollection, the data were entered into a database specific to this study. The data were collected between September and December 2020.

### 2.4. Data Analysis

The exploratory analyses were performed using the free statistical program FACTOR v.10.10.02 (Rovira I Virgili University: Tarragona, Spain) [48], as the data collected were ordinal in nature (4-point Likert scale). Using the Solomon approach [49], the complete sample was divided into two equal subsamples, one for the exploratory factor analysis (EFA), and the other for the confirmatory factor analysis (CFA). The factor extraction was carried out using the robust unweighted least squares (RULS) [50] procedure with Promin rotation [51], presuming a relationship between them. A polychoric correlation matrix [52] was employed due to the nature of the data, and the right number of dimensions was determined by implementing parallel analysis [53]. Normalized Direct Oblimin was chosen as the rotation method for defining factor simplicity and structure after the number of dimensions was determined [54]. As sampling adequacy metrics, the Kaiser–Meyer–Olkin (KMO) and Bartlett tests of sphericity were employed [55,56].

The CFA was then carried out using the software AMOS v.26.0.0 (IBM Corporation, Wexford, PA, USA). Elements having cross loads more than 0.40, communalities less than 0.30, and loads below 0.60 were removed [57]. The following indices were used to evaluate the model’s goodness of fit: (1) the root mean square error of approximation (RMSEA) [58]; (2) the root mean square of residuals (RMSR) [59]; (3) the comparative fit index (CFI) [60]; (4) the non-normed fit index (NNFI) [61]; (5) non-significant values (*p* > 0.05) for the chi-squared probability calculation [62]; and (6) the chi-square per degree of freedom ratio (CMIN/DF) [63]. Additionally, the reliability indices McDonald’s Omega as well as Cronbach’s Alpha were chosen to assess the questionnaire’s final solution [64].

## 3. Results

The explained variance for the first half of the sample, based on eigenvalues [63], was accounted for by four components, which were found using the RULS technique with Promin rotation. Because of the favorable findings provided by the sampling adequacy indices, the exploratory factor analysis was conducted (Bartlett test = 4457.4; df = 153; *p* = 0.000; and KMO test = 0.8128).

Once the number of dimensions was established, the Normalized Direct Oblimin Rotation method was chosen, due to the need for non-parametric approaches because of the degree of kurtosis (kurtosis = 29.369; *p* = 0.000). The rotational loading matrix for 19 variables and four factors is shown in Table 2.

We can see from the rotated loading matrix that there are 19 elements, all of which have loadings greater than 0.3 and are spread across the four previously indicated components. The polychoric correlation matrix from the exploratory analysis is displayed in Table 3.

Item 11 was taken out of the questionnaire after the initial exploratory investigation, since its factor loadings were split between two domains, support (0.377) and community participation (0.362), increasing the downstream analyses’ error rates. Item 18 was also eliminated, since its negative eigenvalues indicated that it was linearly dependent on the other items. Therefore, a factor structure consisting of four dimensions, in turn consisting of 17 items, was extracted.

The structure and factor loadings of each item are shown in Table 4. A factor solution composed of four correlated factors was found: (1) conception of diversity; (2) methodology; (3) support; and (4) community participation.

The association between CEFI-R factors is seen in Table 5.

**Figure 1:**
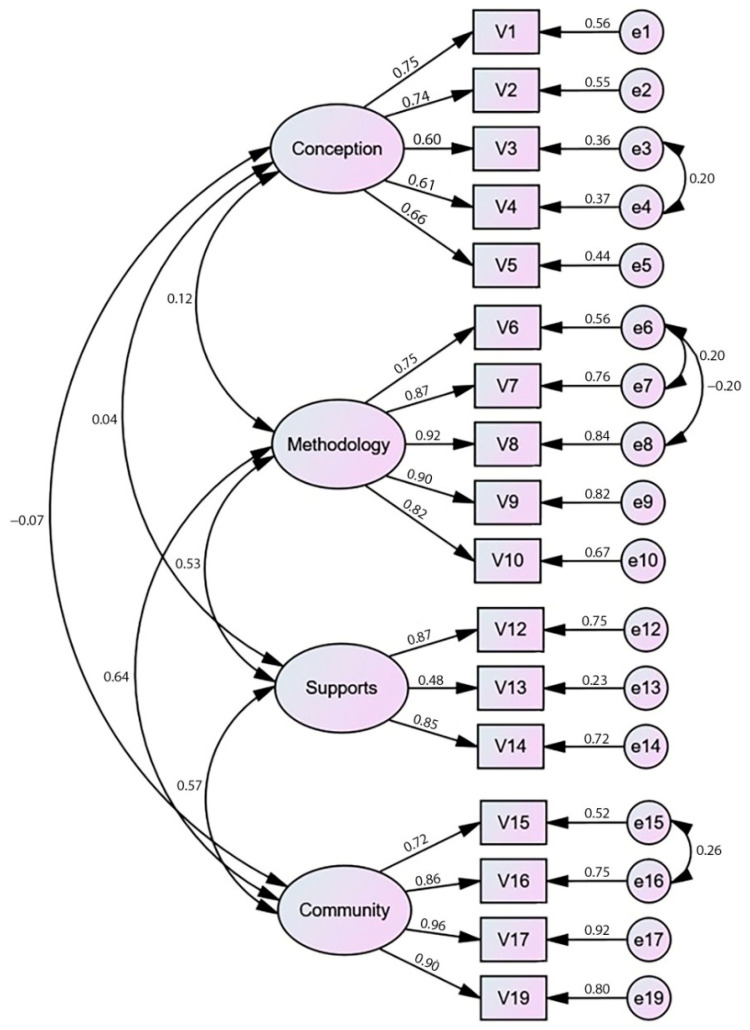
CEFI-R questionnaire factor model.

Once the structure of the questionnaire was defined, CFA was carried out to establish a definitive model (Figure 1) with the other half of the sample.

Following the CFA, Table 6 displays the CEFI-R goodness-of-fit indices, demonstrating a strong fit between the model and the data [65]. The CMIN/DF index shows good values considering it must be below 2 for a correct model fit, and also the chi-squared probability is excellent due to non-significant values. NNFI and CFI over 0.9 mean a near-perfect fit to the model. The RMSEA is within the established limits (0.010–0.050), and the RMSR under 0.08 could be viewed as exceptional.

Table 7 displays the Cronbach’s Alpha and McDonald’s Omega reliability indices for the CEFI-R dimensions, as well as the explained variance for each factor.

## 4. Discussion

This research offers as a final result the psychometric properties, as well as indicators of reliability and validity, of a tool that allows us to characterize the perceptions that teachers in the area of PE have about their training in the area of inclusion. In this way, a final structure was found to be composed of 17 items divided into four inter-related factors, showing excellent goodness-of-fit indices. Similarly, the reliability statistics show values above 0.8, so that the factors can be considered as satisfactory. Therefore, this study provides a simple and easy-to-administer scale to understand perceptions of inclusion training so that public institutions can develop training programs taking into account the current level of teacher education in the field.

Regarding the first dimension, in general, teachers have negative perceptions about inclusion in education, mainly because they do not feel prepared to include pupils with special needs in the sessions [7]. However, in the context of PE, attitudes are very wide-ranging, conditioned mainly by the teacher’s gender, previous experience in inclusive classrooms, level of training, and self-perceived effectiveness [17]. Despite this, there are studies that show that teachers with more experience in inclusive contexts have worse perceptions than their colleagues [21]. Consequently, an urgent need for the reform of training programs has been indicated in order to improve attitudes towards inclusion by increasing teachers’ abilities and willingness to educate learners with special needs [16].

In terms of a teacher’s ability to design and implement inclusive practices in the area of PE, teachers point to training, both initial and in-service, as the fundamental conditioning factor [32,66]. This training deficit is normally coupled with a lack of support infrastructures, inadequate resources, unfamiliarity with specialized equipment, and limited understanding of special educational needs [67], leading to low student participation in the physical activities developed at school [13]. It is therefore suggested that building positive supportive relationships, adapting equipment, activities, and environments, and negotiating differentiated learning experiences’ are supportive pedagogical strategies [68]. In addition, the use of activities based on cooperative learning or peer tutoring seems to have very positive effects [20].

Likewise, the support received in the PE classroom from other teachers is a very interesting topic. For example, strategies such as co-teaching are found to be an effective instrumental and pedagogical model for handling diversity from which students with and without special needs can benefit, allowing the teacher to gain experience in inclusive classrooms in a safer and more secure way [69], as well as the involvement of a support teacher in the classroom [31]. In the same way, peer support can enhance desired behavioral change in a fairly short period of time, as well as improve the social skills of all pupils [18].

Finally, inclusive education implies a shared responsibility among all school team members, and a shared vision with explicit goals towards inclusion can be helpful in its successful implementation [70], as the attitudes of the environment can positively or negatively influence the teacher’s ability to deal with the behavior of learners with special needs [30]. Furthermore, the social participation of these pupils can be classified as essential for their personal development [71], being students open to friendships with special education needs peers [72]. Equally, parental attitudes need to be improved, although they are generally positive towards inclusion [73], as it has been identified that parents of children with special needs are more likely to choose a special school when the child gets older and when the child has severe needs [74].

### 4.1. Practical Implications

Since the beginning of educational inclusion, the strong association between teachers’ perceptions of their training and effectiveness and their attitudes towards inclusion has been noted [75]. However, most of the research in this area of education has focused on instruments that assess in-service teacher effectiveness [76,77], perceptions of their training in inclusion during their higher education [78,79], or qualitative research showing teachers’ perceptions of the implementation of inclusion in the PE session [17,80], expressing doubts about their readiness to implement the inclusion process in the sessions [19].

Therefore, this study presents for the first time in the PE classroom an instrument focused on the analysis of teachers’ training in terms of educational inclusion, thereby analyzing their own perceptions. Therefore, public institutions have at their disposal a validated, free, and easy-to-administer tool that will allow them to design and adapt in-service training courses according to the training demands shown by teachers, as well as to adapt initial teacher training to the reality currently experienced in classrooms. In addition, its use allows teachers and management bodies to identify the educational needs of the centers where they work, so that lines of action and collaboration can be put into practice to guarantee educational inclusion as a fundamental right.

### 4.2. Limitations and Future Lines

Like all research, this study has some limitations. All the respondents carried out their professional activity in the Autonomous Community of Extremadura, so the socio-demographic variables could influence the results of the study. Likewise, the teachers belonged to the area of PE, but no distinction was made between education levels. On the other hand, convenience sampling was carried out for the selection of participants, so there was no randomization in the sampling method. For future lines of research, it could be proposed to extend the validation of this tool to the whole Spanish territory, to differentiate both educational stages to implement the use of this instrument at both educational levels and to equalize the participation rates in both genders, as most of the participants were male.

## 5. Conclusions

This research explores the validity and reliability of a scale aimed at finding out the perceptions of PE teachers regarding their preparation for inclusive education. Both the factor analysis and the confirmatory analysis show a structure composed of 17 items encompassed in four dimensions, presenting both excellent goodness-of-fit indices and good reliability indicators. Therefore, we are faced with a scale that provides a useful, easy, and quick tool for public institutions and teaching professionals to analyze inclusion training.

Teachers’ perception of their readiness for inclusive education is an important part of this fundamental principle, as teachers’ thoughts about their self-efficacy and previous experience are conditioning factors for implementing inclusive practices and strategies in classrooms.

## Figures and Tables

**Table 1:** Sample’s sociodemographic characteristics (N = 789).

Variables	Categories	N	%
Gender	Men	638	80.9
	Women	151	19.1
Center Province	Cáceres	211	26.7
	Badajoz	578	73.3
Studies at University of Extremadura	Yes	54	17.8
	No	78	25.7
Age	Below 30	79	10.0
	Between 30 and 40	253	32.1
	Between 40 and 50	267	33.8
	Over 50	190	24.1

**Table 2:** Rotated loading matrix with Normalized Direct Oblimin.

Items	Factor 1	Factor 2	Factor 3	Factor 4
1. I would prefer to have students with specific educational needs in my classroom.	0.790	0.011	−0.022	0.058
2. A child with specific educational support needs does not disrupt the classroom routine and disrupt the learning of his/her classmates.	0.788	−0.023	0.038	0.050
3. We should place students with special educational needs in mainstream schools even if we do not have the appropriate preparation to do so.	0.604	0.030	−0.060	−0.004
4. Students with specific educational support needs can follow the day-to-day curriculum.	0.708	0.039	0.121	−0.208
5. I am not worried that my workload will increase if I have students with specific educational supports needs in my class.	0.684	−0.029	−0.050	0.108
6. I know how to teach each of my students differently according to their characteristics.	0.003	0.849	−0.011	−0.017
7. I know how to design teaching units and lessons with the diversity of students in mind.	0.022	0.977	−0.053	−0.024
8. I know how to adapt the way I assess the individual needs of each of my students.	0.021	0.975	−0.035	0.014
9. I know how to handle and adapt teaching materials to respond to the needs of each of my students.	0.027	0.922	0.033	−0.003
10. I can adapt my communication techniques to ensure that all students can be successfully included in the mainstream classroom.	−0.046	0.810	0.061	0.070
11. Joint teacher-support teacher planning would make it easier for support to be provided within the classroom.	−0.077	0.290	0.377	0.362
12. I believe that the best way to provide support for students is for the support teacher to be embedded in the classroom, rather than in the support classroom.	−0.006	0.047	0.709	0.165
13. The role of the support teacher is to work with the whole class.	0.021	−0.005	0.494	0.011
14. I consider that the place of the support teacher is in the regular classroom with each of the teachers.	−0.026	−0.012	1.004	−0.033
15. The educational projects should be reviewed with the participation of the different agents of the educational community (teachers, parents, students).	0.071	−0.028	0.204	0.702
16. There must be a very close relationship between the teaching staff and the rest of the educational agents (AMPA, neighbourhood associations, school council…).	0.027	−0.042	−0.009	0.979
17. The school must encourage the involvement of parents and the community.	0.006	0.046	−0.027	0.951
18. Each member of the school (teachers, parents, students, other professionals) is a fundamental element of the school.	−0.053	0.154	0.097	0.837
19. The school must work together with the resources of the neighbourhood.	−0.001	0.055	0.006	0.908

**Table 3:** Polychoric correlation matrix extracted from the EFA.

Item	1	2	3	4	5	6	7	8	9	10	11	12	13	14	15	16	17	18	19
1	1.00																		
2	0.65	1.00																	
3	0.44	0.497	1.00																
4	0.51	0.56	0.46	1.00															
5	0.58	0.48	0.41	0.49	1.00														
6	0.12	0.08	0.03	0.10	0.09	1.00													
7	0.14	0.09	0.11	0.10	0.12	0.85	1.00												
8	0.14	0.18	0.08	0.06	0.10	0.78	0.92	1.00											
9	0.15	0.14	0.09	0.09	0.10	0.76	0.84	0.94	1.00										
10	0.11	0.06	0.06	−0.01	0.06	0.72	0.79	0.82	0.83	1.00									
11	0.02	0.09	−0.01	−0.12	0.03	0.48	0.54	0.61	0.58	0.65	1.00								
12	0.07	0.08	−0.04	−0.01	0.08	0.32	0.32	0.37	0.44	0.43	0.75	1.00							
13	0.04	0.05	0.01	0.06	−0.01	0.17	0.18	0.18	0.18	0.23	0.26	0.40	1.00						
14	0.02	0.05	−0.02	0.05	0.02	0.28	0.31	0.34	0.36	0.38	0.67	0.79	0.51	1.00					
15	0.13	0.13	−0.01	0.09	0.43	0.40	0.42	0.42	0.42	0.64	0.55	0.32	0.39	0.61	1.00				
16	0.08	0.06	0.02	−0.07	0.11	0.42	0.43	0.49	0.47	0.50	0.68	0.58	0.30	0.57	0.80	1.00			
17	0.08	0.06	0.01	−0.06	0.08	0.44	0.51	0.55	0.54	0.55	0.71	0.59	0.32	0.55	0.76	0.92	1.00		
18	0.03	0.11	−0.05	−0.09	0.03	0.51	0.57	0.61	0.62	0.63	0.83	0.69	0.30	0.63	0.81	0.90	0.94	1.00	
19	0.03	0.08	0.03	−0.06	0.07	0.43	0.49	0.53	0.55	0.57	0.71	0.61	0.31	0.55	0.77	0.88	0.91	0	1.00

**Table 4:** CEFI-R questionnaire rotated factor solution and factor loading.

Items	Factor 1	Factor 2	Factor 3	Factor 4
1. I would prefer to have students with specific educational needs in my classroom.	0.788			
2. A child with specific educational support needs does not disrupt the classroom routine and disrupt the learning of his/her classmates.	0.788			
3. We should place students with special educational needs in mainstream schools even if we do not have the appropriate preparation to do so.	0.603			
4. Students with specific educational support needs can follow the day-to-day curriculum.	0.711			
5. I am not worried that my workload will increase if I have students with specific educational supports needs in my class.	0.682			
6. I know how to teach each of my students differently according to their characteristics.		0.849		
7. I know how to design teaching units and lessons with the diversity of students in mind.		0.977		
8. I know how to adapt the way I assess the individual needs of each of my students.		0.977		
9. I know how to handle and adapt teaching materials to respond to the needs of each of my students.		0.925		
10. I can adapt my communication techniques to ensure that all students can be successfully included in the mainstream classroom.		0.814		
11. Joint teacher-support teacher planning would make it easier for support to be provided within the classroom.	Excluded			
12. I believe that the best way to provide support for students is for the support teacher to be embedded in the classroom, rather than in the support classroom.			0.727	
13. The role of the support teacher is to work with the whole class.			0.489	
14. I consider that the place of the support teacher is in the regular classroom with each of the teachers.			1.004	
15. The educational projects should be reviewed with the participation of the different agents of the educational community (teachers, parents, students).				0.679
16. There must be a very close relationship between the teaching staff and the rest of the educational agents (AMPA, neighbourhood associations, school council…).				0.977
17. The school must encourage the involvement of parents and the community.				0.930
18. Each member of the school (teachers, parents, students, other professionals) is a fundamental element of the school.	Excluded			
19. The school must work together with the resources of the neighbourhood.				0.868

**Table 5:** CEFI-R questionnaire inter-factor correlation matrix.

	Factor 1 Conception of Diversity	Factor 2 Methodology	Factor 3 Support	Factor 4 Community Participation
Factor 1 Conception of Diversity	1			
Factor 2 Methodology	0.135	1		
Factor 3 Support	0.049	0.404	1	
Factor 4 Community Participation	0.038	0.542	0.625	1

**Table 6:** CEFI-R questionnaire goodness-of-fit indices.

Indices	Value
RMSEA	0.045
RMSR	0.039
NFI	0.956
NNFI	0.980
*Ρ (χ* ^2^ *)*	0.99
CMIN/DF	1.803

RMSEA: root mean square error of approximation; RMSR: root mean square of residuals; NNFI: non-normed fit index; CFI: comparative fit index; *Ρ (χ*^2^*)*: chi-squared probability; CMIN/DF: minimum discrepancy per degree of freedom.

**Table 7:** Internal consistency of the CEFI-R questionnaire.

	Factor 1 Conception of Diversity	Factor 2 Methodology	Factor 3 Supports	Factor 4 Community Participation
Cronbach’s Alpha	0.803	0.934	0.807	0.923
McDonald’s Omega	0.812	0.935	0.815	0.924
Explained Variance	2.597	4.439	2.288	3.556

## Data Availability

The datasets used during the current study are available from the corresponding author on reasonable request.

## Appendix A

Cuestionario para la Evaluación de la Preparación del Profesorado para la Inclusión (CEFI-R).

1 Preferiría no tener en mi aula alumnos con necesidades específicas de apoyo educativo
2 Un niño con necesidades específicas de apoyo educativo interrumpe la rutina del aula y perjudica el aprendizaje de sus compañeros
3 No debemos escolarizar alumnos con necesidades educativas especiales en centros ordinarios hasta que no tengamos la formación adecuada para ello
4 Los alumnos con necesidad específica de apoyo educativo no pueden seguir el día a día del curriculum
5 Me preocupa que mi carga de trabajo se incremente si tengo alumnos con necesidades específicas de apoyo educativo en mi clase
6 Sé cómo enseñar a cada uno de mis alumnos de manera diferente en función de sus características individuales
7 Sé cómo elaborar las unidades didácticas y las clases teniendo presente la diversidad de los estudiantes
8 Sé cómo adaptar mi forma de evaluar a las necesidades individuales de cada uno de mis alumnos
9 Sé cómo manejar y adaptar los materiales didácticos para responder a las necesidades de cada uno de mis alumnos
10 Soy capaz de adaptar mis técnicas de comunicación para asegurarme de que todos los alumnos puedan ser incluidos con éxito en el aula ordinaria
11 La planificación conjunta profesor-profesor de apoyo facilitaría que los apoyos se proporcionaran dentro del aula
12 Creo que la mejor manera de proporcionar apoyo a los alumnos es que el profesor de apoyo se incorpore al aula, en lugar de hacerlo en el aula de apoyo
13 La función del profesor de apoyo es trabajar con todo el alumnado de mi aula
14 Considero que el lugar del profesor de apoyo está dentro del aula ordinaria con cada uno de los profesores
15 El proyecto educativo debería revisarse con la participación de los distintos agentes de la comunidad educativa (profesores, padres, alumnos…)
16 Es fundamental que haya una relación muy estrecha entre el profesorado y el resto de agentes educativos (AMPA, asociación de vecinos, consejo escolar…)
17 La escuela debe fomentar la implicación de los padres y de la comunidad
18 Cada miembro del centro educativo (profesores, padres, alumnos, otros profesionales) es un elemento fundamental del mismo
19 El centro debe trabajar de forma conjunta con los recursos del barrio (biblioteca, servicios sociales, servicios sanitarios…)
